# Adverse Childhood Experiences (ACEs) and Environmental Exposures on Neurocognitive Outcomes in Children: Empirical Evidence, Potential Mechanisms, and Implications

**DOI:** 10.3390/toxics11030259

**Published:** 2023-03-10

**Authors:** Margaret Gladieux, Nathan Gimness, Bianca Rodriguez, Jianghong Liu

**Affiliations:** Department of Family and Community Health, School of Nursing, University of Pennsylvania, Philadelphia, PA 19104, USA

**Keywords:** adverse childhood experiences (ACEs), neurocognition, socioeconomic status (SES), environmental exposure, toxicants

## Abstract

The purpose of this article is to examine the current literature regarding the relationship between adverse childhood experiences (ACEs) and environmental exposures. Specifically, the paper will focus on how this relationship between ACEs and physical environmental factors impacts the neurocognitive development of children. With a comprehensive literary search focusing on ACEs, inclusive of socioeconomic status (SES), and environmental toxins common in urban environments, the paper explores how these factors contribute to cognitive outcomes that are associated with the environment and childhood nurturing. The relationship between ACEs and environmental exposures reveals adverse outcomes in children’s neurocognitive development. These cognitive outcomes include learning disabilities, lowered IQ, memory and attention problems, and overall poor educational outcomes. Additionally, potential mechanisms of environmental exposures and children’s neurocognitive outcomes are explored, referencing data from animal studies and evidence from brain imaging studies. This study further analyzes the current gaps in the literature, such as the lack of data focusing on exposure to environmental toxicants resulting from experiencing ACEs and discusses the research and social policy implications of ACEs and environmental exposure in the neurocognitive development of children.

## 1. Introduction

The negative impacts of environmental toxicants and exposures on childhood neurocognitive development have been documented and show increasing evidence that a wide range of environmental toxicants have significant influence on children’s cognitive development; these include air pollution [1,2] and lead [3,4]. Moreover, factors that can lead to adverse childhood experiences (ACEs), such as socioeconomic status [5,6] and poor neighborhood living conditions [7], are shown to contribute to children’s poor cognitive outcomes and exposure to these environmental toxicants. Adverse childhood experiences (ACEs) are events in childhood that are potentially traumatizing and create a lasting impact on one’s physical and emotional health [8]. ACEs are designated into three categories: abuse (emotional, physical, and sexual abuse), household challenges (exposure to parental violence, household substance abuse, mental illness, parental separation, and household members who have gone to prison), and physical or emotional neglect [9]. According to recent literature, low socioeconomic status (SES) is so closely tied with ACEs that low SES itself can be considered as an adverse childhood experience within the categorization of being a household challenge. Some studies have previously examined ACEs and SES separately or examined SES as a mediating factor, linking inaccessibility of resources to negative health consequences. However, more recent studies, especially in light of the COVID-19 pandemic, have considered ACEs inclusive of SES, rather than examining them as separate factors; this is due to how low SES is so often co-morbid with high ACEs and can be considered a negative household experience, a category of ACEs [10,11]. For instance, in literature examining the currently defined ACEs scale, low socioeconomic status is classified as an impactful form of trauma consistent among various racial and ethnic groups, classifying it as an ACE [12]. However, the majority of research has focused on ACEs and socioeconomic status in a mutually exclusive manner, rather than together. In this review, we adhere to a categorization of ACEs that includes low SES as a potential ACE. The current review focuses on evidence demonstrating that ACEs, including socioeconomic status, exacerbate the effect of exposure to environmental toxicants and negatively affect neurocognitive development. Resulting negative cognitive outcomes include lowered IQ, difficulties with memory and attention, and learning disabilities. 

Exposure to environmental factors, such as polluted air and lead, has been correlated with negative neurocognitive outcomes in children [13,14]. Air pollution has been associated with higher instances of learning disabilities [15,16,17], lower IQ [18], problems with working memory [18], attention deficits [19], impaired academic performance [20], and decreased cortical volume in children [21]. Lead exposure is also known to interrupt cognitive development and is associated with poor executive function [22], ADHD symptoms [23], and lowered IQ scores [24]. Additionally, ACEs including socioeconomic status, parental education, and neighborhood conditions all have implications on children’s cognitive development; it is important to consider these social factors in conjunction with the physical environments that encompass them in considering the effects they have on cognitive outcomes [25,26].

The primary purpose of this paper is to examine the relationship between ACEs and environmental toxicants in negatively impacting cognitive development. Special attention is given to the mediated effects of low socioeconomic status and air pollution, lead and neighborhood conditions, and other sources of environmental toxicity in the household. Some of the major findings examined in this paper include the ways in which ACEs, including low socioeconomic status, exacerbates risk of exposure to toxins such as air pollution and lead, as well as ways in which this creates a lasting impact in cognitive development. Discussions for potential mechanisms of action for each toxin are also included and depicted in Table 1. This discussion is followed by an analysis of potential policy implications and current gaps in the literature.

## 2. Methods

This narrative literature review encompasses recent literature examining the relationship between ACEs, sources of environmental toxicity, and cognitive outcomes. Using PubMed, PsycInfo, Web of Science, and Google Scholar, we conducted a search that considered adverse childhood experiences inclusive of socioeconomic status and cross-sectional and longitudinal studies of neurocognitive outcomes. Specifically, we focused on papers published that considered both socioeconomic factors and physical environmental factors and their effects on cognition, behavior, and academic performance. 

We began by narrowing outcomes to those closely tied to environmental factors (IQ, working memory, cognition, academic performance, ADHD symptoms) and excluding outcomes that have strong evidence-based genetic components (such as autism or other neurodevelopmental disorders). In considering our search criteria for ACEs, we were inclusive of low SES within ACEs; in preliminary literature searches, we found that very few studies consider solely ACEs, environmental toxic exposures, and outcomes; including SES provided a more robust pool of literature from which to consider the multidimensional effects of socioeconomic adversity and adverse environment factors.

**Figure 1:**
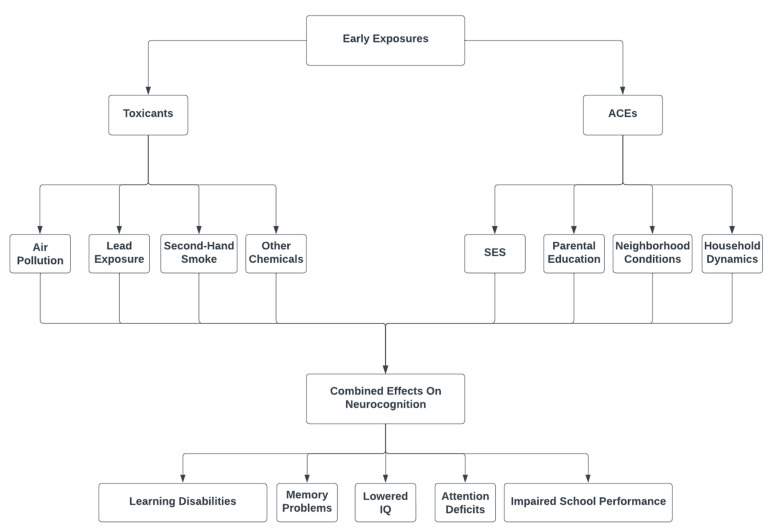
Model of Combined Effects of ACEs and Environmental Toxicants on Neurocognition.

The model in Figure 1 demonstrates that childhood early exposure to environment toxicants and ACEs may result in poor neurocognition due to the compounding effect of toxicants and ACEs. These toxicants include air pollution, lead exposure, second-hand smoke, and other chemicals. ACEs include low SES, low parental education, poor neighborhood conditions, as well as dysfunctional family dynamics. There are many indicators of neurocognition, including learning disabilities, memory problems, lowered IQ, attention deficits, and impaired school performance. While this model emphasizes the combined effect of toxicants and ACE exposure on neurocognition, it must also be noted that environmental exposures can be mediating factors in the relationship between ACEs and cognitive development, which means ACEs may predispose children to be more vulnerable to environmental toxicant exposure, which further results in cognitive deficits. This concept has been demonstrated in a recent study, where environmental toxicant (blood lead) is positively linked to more social adversity and behavioral problems, and blood lead levels mediated the social adversity–behavior relationship in children [27]. The finding also emphasizes the importance of both social and environmental determinants of adolescent health and highlights the need to mitigate adverse social influences and monitor lead exposure in children’s environments to reduce the likelihood of developing problems with externalizing behaviors. While the mediating role of lead exposure in social adversity–behavior has been reported, future research on mediating factors of toxicants in the relationship of ACEs and neurocognition is greatly needed.

## 3. ACEs and Air Pollution

A child’s exposure to air pollution is connected to adverse childhood experiences, specifically growing up with low socioeconomic status. Low socioeconomic status increases one’s risk of exposure to air pollution. For example, there are greater levels of air pollution in urban cities, areas where low socioeconomic status populations have a greater tendency to live [28]. Socially disadvantaged communities are at greater risk of exposure to air pollution because of proximity to industrial sites and poor air filtration [29]. Current studies show that air pollution is a particular environmental concern for children’s cognitive development. Exposure to air pollution, including particulate matter (PM10) and polycyclic aromatic hydrocarbons (PAHs), is associated with several negative cognitive outcomes including higher rates of learning disabilities, lowered IQ scores, memory and attention deficits, and overall issues with school performance [30].

Childhood exposure to air pollution combined with adverse socioeconomic conditions has been associated with higher rates of adverse cognitive outcomes in children. A geographic study of representative communities indicated that areas with higher levels of air pollution and lower socioeconomic status have a larger occurrence of learning disabilities within their youth populations than those from more affluent backgrounds and less air pollution in their community [31]. In another study using geographic models, higher rates of air pollution were found in more socioeconomically disadvantaged neighborhoods [32]. This pattern was then matched with the spatial concentrations of children with learning disabilities. The highest rates of childhood learning disabilities were concentrated in the areas with both high air pollution and low socioeconomic status [32]. In a separate study, ADHD was more prevalent in boys living under lower socioeconomic status and they had greater exposure to particulates of air pollution [17]. These studies indicate that the combined social disadvantages and exposure to air pollution compound risk for neurocognitive impairments and learning disabilities.

These adverse cognitive effects from air pollution are limited not only to exposure in childhood, but also extend to prenatal exposure. Additional studies show that regardless of socioeconomic status, poor cognitive outcomes are associated with air pollution exposure, even with prenatal exposure during early neurodevelopment [1]. In a cohort study that followed a group of children prenatally to age 9, high levels of prenatal exposure to PAH in the air resulted in more ADHD symptoms in childhood; this difference was more pronounced in children who experienced persistent hardship during childhood [33]. Prenatal PAH exposure predicted ADHD symptoms, but these symptoms were more severe in children who had also experienced persistent hardship in addition to air pollution exposure. Similarly, prenatal exposure to air pollution is also implicated in lowered IQ scores. Children exposed to prenatal PM10 scored on average 2.5 points lower on IQ tests than children with no air pollution exposure; children with mothers who had low plasma folate, an indicator of poor nutrition and a proxy indicator for low parental education and socioeconomic status, in addition to exposure to prenatal air pollution, averaged 6.8 points lower than any other group [34]. These cognitive impacts associated with co-morbidity of prenatal air pollution exposure and social adversity have been demonstrated not only with IQ, but also general measures of working memory and attention. Significant inverse effects of high cord PAH–DNA adducts on full scale IQ, perceptual reasoning, and working memory scores were observed in the groups whose mothers reported a high level of material hardship during pregnancy or recurring high hardship into the child’s early years, and not in those without reported high hardship [35].

Impaired cognition, including attention difficulties, IQ deficits, and working memory problems associated with the combined effects of air pollution and social adversity, is also implicated in applied learning outcomes for children, playing a particular role in educational outcomes. Exposure to air pollution has been demonstrated to have negative impacts on school performance in children with exposure, with more extreme effects in children of lower socioeconomic status [36,37]. These negative outcomes are also associated with externalizing behavior issues, a known outcome of exposure to air pollution in childhood [34,38], which can further disrupt learning. For example, in a 2020 study, third graders with exposure to particulate matter had lower proficiency in both math and English language arts than those without exposure to air pollution in their home neighborhood [36]. These effects were more pronounced in children of lower socioeconomic status. 

Although air pollution has been linked to several negative cognitive outcomes in children and children from disadvantaged backgrounds are more likely to be exposed to air pollution, data about the combined effects of air pollution and specific adverse childhood experiences are limited. Few studies have studied the combined, cumulative effects of socioeconomic adversity and air pollution. Of the studies that have examined the combined effects of social factors and air pollution, most are limited and only provide preliminary results demonstrating the severity of both the effects of air pollution and adversity stemming from ACEs. Additionally, few studies have examined individual social adversity factors, such as parental education and neighborhood conditions, that are most associated with cognitive outcomes when combined with air pollution exposure. Future research is warranted to further understand the interaction effects of different forms of air pollution and factors of social adversity.

## 4. Potential Mechanisms of Action

The underlying mechanism responsible for the relationship between neurocognitive outcomes in children and exposure to air pollution due to ACEs remains unclear. While low socioeconomic status as an ACE directly increases a child’s exposure to air pollution, the link to neurocognitive outcomes from this toxicant requires further study. Possible mechanisms at the molecular level that have been proposed are related to two explanations: neuroinflammation stemming from reductions in cellular epithelial barriers and the emergence of white matter hyperintensities (WMHs).

The erosion of cells in the epithelial layers, due to toxicants, of the lungs, nasal passages, and gut allows for greater entry of toxic particles found within air pollution to enter the body [39]. There is further erosion of cells in the blood–brain barrier, which is detrimental for young children, as it allows for air pollutants to come into contact with the developing brain [39]. It is understood that these pollutants then cause significant neuroinflammation to neurons within the brain and the central nervous system, leading to cognitive impairments [39]. As evidence, diesel exhaust particles found in air pollution have been found to be associated with neuroinflammation in mice, the accumulation of tau protein associated with Alzheimer’s pathology, and the degradation of specific tight junction proteins in the capillaries of the blood–brain barrier, which alters its permeability [39]. The results of this evidence were determined to be analogous with those seen in children and they most significantly affected the prefrontal and frontal cortices, the hippocampus, and olfactory bulb [39]. Additional evidence further suggests that inflammation in the brain, stemming from air pollution, significantly impairs cognitive development. Studies show that oxidative stress, induced by low doses of ozone found as a secondary pollutant in urban smog, impairs the inflammatory processes of the brain [40]. The result of this impairment is progressive neurodegeneration and loss of some brain functionality in the hippocampus [40]. 

In addition to neuroinflammation, the emergence of white matter hyperintensities (WMHs), brain regions with demyelinated neurons that have an impaired ability to communicate with other parts of the brain, has also been associated with impaired neurocognitive outcomes in children [39]. These white matter hyperintensities have been observed through magnetic resonance imaging studies and indicate damaged cytoarchitecture [41]. Children in urban Mexico City, who were exposed to air pollutants, exhibited altered brain volumes and increased white matter volume in frontal and temporal cortices [41]. These increased volumes of white matter indicate greater demyelination of neurons in the brain and central nervous system, which are linked to cognitive impairments in the developing brain of children.

In summary, there is evidence for overall cognitive impairment due to air pollution, but the exact mechanisms require greater study and analysis, as well as more evidence that comprehensively differentiates how children who experience ACEs and have greater exposure to air pollution suffer from cognitive impairments.

## 5. ACEs and Lead Exposure

Adverse childhood experiences and lead exposures are interrelated, and lead to negative outcomes in children. Adverse childhood experiences contribute to the increased possibility that a child may be exposed to lead. Different studies have attributed this to maternal smoking [42], direct ingestion [43], or exposure to lead-based paints [44]. Moreover, exposure to stress during embryonic development has been found to exacerbate lead exposure [45]. Physical neglect is also attributed to a child’s exposure to lead-based paints later in life [46]. 

A poor social environment combined with lead exposure has cumulative effects on the cognitive deficits that are associated with each environmental exposure. Exposure to lead has been shown to be more common in families and children with low socioeconomic status (SES) due to the high probability that they live in areas with older water pipe systems [47]. Lead exposure can also be the result of interactions with urban soils [48], which disproportionally affect people of low SES [49]. One study has attributed this increased exposure to lack of education [50]. Another study attributes increased exposure to the proximity of families to industrial sites in lower SES neighborhoods [51]. This is of concern because cognitive deficits associated with lead exposure and adversity are not limited to childhood. In fact, the effects of these cumulative exposures can span into adulthood. In childhood, postnatal lead exposure is associated with lowered IQ across the lifespan. In a longitudinal study of New Zealanders, lead exposure in childhood was significantly associated with lower cognitive function and socioeconomic status at the age of 38 years; greater childhood lead exposure was also associated with greater declines in IQ from childhood to adulthood and greater declines relative to parents in occupational socioeconomic status [4]. The long-term effect of lead exposure on overall cognition is not only a concern because it disproportionately affects people from lower socioeconomic status, but because it directly impedes social mobility by decreasing cognitive ability. 

Beyond generalized cognitive outcomes associated with lead exposure and adversity stemming from low socioeconomic status and ACEs, lead exposures both prenatally and postnatally are associated with several specific cognitive deficiencies, including lowered IQ and impaired working memory. Even before childhood exposure, prenatal lead exposure is associated with cognitive deficits in childhood. Lead is extremely toxic to the developing brain; in a 2017 study, children exposed to prenatal lead had lower IQ scores at both 4 and 8 years of age than children with no lead exposure, indicating that early exposures to lead have negative effects on cognitive development [3]. Working memory is also affected by ACEs and lead exposure; in a study of a rural and impoverished community in Malaysia, elementary schoolers who were in proximity to a former mine demonstrated significant deficits in working memory capacity [52]. In another study of the China Jintan cohort, children with blood lead exposure beginning at ages of 3–5 years demonstrated reduced working memory abilities in adolescence [53].

Despite these links between cognitive outcomes and the cumulative effects of lead exposure and social adversity, the field of study is still limited. Many studies treat adversities stemming from ACEs as covariates rather than variables. Thus, there is limited knowledge about the exact mechanism underlying the magnifying effects between lead exposure and social adversity. For example, ADHD is strongly associated with lead exposure, especially in vulnerable populations, but because social adversity is also a risk factor for ADHD, it is often treated as a confounding variable rather than a mechanism for magnification [54]. Further research is needed for more comprehensive analysis of these cumulative effects.

## 6. Potential Mechanism of Action

The precise mechanism underlying the relationship between neurocognitive outcomes in children and lead exposure due to ACEs is not completely understood. It has been observed that children with ACEs, including poor maternal care and neglect, suffer from increased lead exposure and its detrimental effects. While ACEs have been shown to increase a child’s exposure to lead, the associated neurocognitive outcomes of exposure to this toxicant require additional study. Possible mechanisms at the molecular level that have been proposed are related to at least three explanations: impeding synaptic transmission through the mimicking of calcium, the reduction of NMDA receptors, and phosphorylation of protein kinase C (PKC).

Lead is known to compromise the neural wiring of the brain by impeding synaptic transmission and mimicking the action of calcium in cellular processes. Experimental studies show that lead increases the frequency of mini end plate potentials (MEPPs) in presynaptic nerve endings by either substituting for calcium or galvanizing intracellular stores of calcium [55]. The growing frequency of MEPPs caused by lead results in the increasingly spontaneous release of neurotransmitters from the presynaptic nerve terminals, which impairs the structural wiring of the developing brain [55]. 

Animal studies have also indicated that lead exposure during early synaptic brain development reduces the content of NMDA receptors with NR1/NR2A subtypes that are in presynaptic proximity to the protein synaptophysin [56]. These studies further show that the effects of lead exposure reflect those of the NMDA receptor antagonist APV [56]. The inhibition of these receptors by lead is detrimental for neurocognitive development as NMDA receptors are crucial for the processes of learning and memory at the physiological level [57].

Additional animal studies have implicated lead’s inhibitory effects in impairing neurocognition PKC. Based on the available evidence, these studies have proposed that acute exposure of neurons to lead is sufficient for the activation of PKC in the hippocampus, which phosphorylates nicotinic acetylcholine receptors (nAChRs) [58]. The phosphorylation of this receptor and proteins associated with it inactivates synaptic transmission and disrupts memory, learning, and other neurocognitive processes [58]. 

In summary, there are many proposed mechanisms for how lead affects neurocognitive development and outcomes in children and adults, but many rely on inferences made from animal studies as it is difficult to directly study the effects of lead on humans. Additionally, there are many competing mechanisms to isolate the specific mechanisms by which lead impairs the brain, but research continues to reveal the necessity for more studies and analysis of lead exposure.

## 7. Other Sources of Environmental Toxicity

In addition to air pollution and lead exposure, there are other sources of environmental toxicity that lead to negative cognitive outcomes in children, particularly exposure to chemical pesticides, household chemicals, and environmental tobacco. These environmental toxicants are known to cause several developmental problems in children, and children from socially disadvantaged communities are particularly susceptible [59]. Studies show that the most likely routes of exposure to these toxic substances are in children’s households [60]. Unhealthy neighborhood conditions and low socioeconomic positions also act as a route of exposure; in a publication on the intersection of poverty and environmental exposures, it was found that a lack of proper housing will create greater exposure to neighborhood toxicants [61].

Chemical pesticides are common neurotoxicants in children. Exposure to chemical pesticides has been demonstrated to cause deficits in language development and cognitive performance in very young children; in one study of 190 low-income mothers and their young children, exposure to toxicants, especially pesticides, was reported by about 20% of mothers during or around pregnancy, and 30% when their children were between 1 and 2 years of age [62]. Toxicant exposure was significantly associated with lags in language and cognition even when controlling for socioeconomic factors [62]. Additional studies have shown that adverse effects are more pronounced in children with higher levels of social adversity; negative associations between dialkyl phosphate concentrations and IQ were stronger in children experiencing greater adversity [63]. In a study of low-income Latina mothers and their children in the Salinas Valley, total adversity and specific domains of adversity, including poor learning environment and adverse parent–child relationships, were negatively associated with child cognition [63].

Tobacco exposure is another common toxicant which children from more disadvantaged backgrounds and with exposure to ACEs are more likely to encounter. Parents from lower socioeconomic backgrounds and with less education are less likely to be informed on the dangers of second-hand tobacco smoke on children’s health and development, and thus children from lower socioeconomic backgrounds are more likely to be exposed not only within their households [64], but also prenatally during their mother’s pregnancy [65]. In a study of 4–15 year olds, children who had encountered either prenatal tobacco exposure or environmental tobacco exposure were more likely to have parent-reported learning disabilities [66]. Covariates included in the analysis were age, race, gender, care in an NICU, attendance at a preschool or daycare, and socioeconomic status. Children who had not attended preschool and those with lower socioeconomic status also had more prevalence of learning disabilities [67].

A final common neurotoxin affecting children’s cognitive development is household chemicals such as polybrominated diphenyl ethers (PBDEs), chemicals commonly found in consumer products. Both prenatal and childhood exposures are associated with several negative cognitive outcomes in children and adolescents [68]. There is a significant correlation between PBDE exposure and socioeconomic status. Parental education, household cleaning habits, material hardship, and second-hand tobacco smoke exposure are all predictors of higher cord PBDE levels, indicating prenatal exposure [69]. Prenatal PBDE exposure is implicated in lowered IQ scores of up to four points in early childhood [68]. PBDE has demonstrated negative impacts on memory systems, with children with high PBDE levels testing up to 8 points lower on memory tasks than children with lower levels or no exposure [70]. Mitigating household exposure to neurotoxicants is a vital public health concern as it more pronouncedly and negatively affects vulnerable communities where children experience more adversity.

## 8. Potential Mechanism of Action

There are multiple potential mechanisms that have been proposed to explain the relationship between neurocognitive outcomes in children and exposure to pesticides, PBDEs, and tobacco due to ACEs. It has been shown that children with ACEs often suffer from and are exposed to poorer quality of life due to low socioeconomic status and these ACEs are thus associated with a child’s increased exposure to these toxicants. However, the correlated neurocognitive outcomes due to exposure to these toxicants require further study as the mechanisms of action are not completely understood. Possible mechanisms at the molecular level that have been proposed are related to the following: pesticides inhibiting acetylcholinesterase [71], household chemicals (PBDEs) disrupting the regulation of thyroid hormones [72], and the methylation of DNA in the placenta of infants due to exposure to cigarette smoking, stemming from maternal neglect, and tobacco [73].

It has been observed that pesticides, primarily organophosphorus, impact the neurocognition of children. One specific mechanism shows that a decrease in cholinergic neurons is responsible for the toxicity that chemical pesticides have in neurodevelopment. These pesticides inhibit acetylcholinesterase, an enzyme responsible for the hydrolysis of the neurotransmitter acetylcholine into choline and acetic acid [71]. The organophosphorus pesticides attach to the enzyme by phosphorylation at its active site [71]. When acetylcholine cannot be hydrolyzed, the neurotransmitter accumulates at synaptic clefts throughout the brain and central nervous system, causing dispersed and prolonged depolarization of cholinergic neurons [71]. It is this prolonged stimulation of cholinergic neurons that results in neurocognitive impairment because of pesticide exposure.

Likewise, it is shown that household chemicals such as polybrominated diphenyl ethers (PBDEs) affect neurocognition at the molecular level. The primary mechanism of action of PBDE neurotoxicity was evaluated by analyzing thyroid hormone disruption [72]. This comparison is rationalized due to the structural similarities between thyroxine (T4) and PBDEs [72]. Data from animal studies have shown that deficits and excesses in thyroid hormones cause abnormal growth of dendrites, axons, and cells of the cerebellum [72]. Moreover, studies have shown associations between PDBE exposure in children with greater amounts of thyroid hormone T3 [72]. How these excesses and deficiencies of T4 or PBDE precisely affect molecular or cellular targets to cause these abnormalities remains unknown; however, neuronal apoptosis has been observed in response to oxidative stress from PBDE exposure [72]. 

The effects of tobacco exposure on neurodevelopment additionally have a specific and prenatal proposed mechanism of action. Studies have detected atypical DNA methylation in the placenta of infants who have been exposed to cigarette smoke and tobacco [73]. 

In summary, there remains a lack of complete clarity as to how each of these toxicants’ modes of action affect the developing brain in children who suffer from exposure due to ACEs. However, the evidence and research suggest that these toxicants have an overwhelmingly impairing effect on neurocognitive outcomes in these children.

## 9. Implications and Conclusions

The government and greater societal institutions have a significant role in mitigating the ways by which ACEs and the environment contribute to the neurocognitive development of children. The large socioeconomic gap in the United States greatly contributes to adverse childhood experiences associated with exposure to poverty and other consequences of having a low socioeconomic status [74]. Furthermore, inconsistent environmental laws currently in place allow for unhealthy levels of pollutants to be generated, especially in urban areas, that exacerbate the exposure of children to toxicants in the environment [75]. However, there are solutions which can alleviate some of the symptoms of carcinogens and toxicant exposure among children. Recent studies have emphasized the importance of access to green spaces for children’s health and development [76,77]. Access to healthy green spaces among children has been demonstrated to mitigate the effects of negative social environments due to ACEs as well as the negative effects of pollutants [78]. Urban green spaces tend to be less available and accessible to those of lower socioeconomic status [79], but the mechanism of action of green space exposure for mitigating the effects of environmental toxicity and ACEs consists of improved emotional health and cognitive function in children [76,77].

This review has demonstrated that environmental toxicants and ACEs collectively contribute negatively towards neurocognitive development in children, including lowered IQ, memory retention issues, and overall poorer educational outcomes. This conclusion is attained from multiple studies and research designs that include longitudinal cohorts and geographic population models. The evidence supports positive associations between ACEs and various environmental exposures, including metals such as lead, air pollution, second-hand smoke, and pesticides, in detrimentally impairing the neurocognitive development of children.

However, substantial research is still needed to highlight the relationship between ACEs and environmental toxicants collectively contributing to impairment in children’s neurocognition [80]. Much of the current literature focuses on their separate effects or takes adversity factors stemming from ACEs as covariates as opposed to actual variables in addition to the environment. Moreover, there is a need to consider a broader classification of ACEs that includes the effects of poverty and low socioeconomic status as adverse childhood experiences. The evidence provided by current research suggests there is still a dearth of knowledge regarding a complete understanding of the effects of these variables on the development of children and thus necessitates more comprehensive research and environmental policy changes. The underlying biological mechanisms of action have been substantially studied but many of the precise means of action of these environmental variables are still relatively unknown. Further research is needed to illustrate these pathways linking environmental exposures, ACEs, and impaired neurocognition. Despite great amounts of research and studies that have been conducted demonstrating the detrimental health outcomes related to these exposures, much more effort and significant policy changes are needed to mitigate the harmful effects of environmental factors and ACEs on children. The impairment children experience during early neurocognitive development can contribute to health effects that persist for the entirety of their lives and their health should thus be prioritized in policymaking regarding socioeconomic policy changes and environmental regulations.

## Figures and Tables

**Table 1:** Summary of Sources, Mechanisms of Toxicity, and Outcomes for Toxicants.

Toxicant	Exposure Source	Mechanistic Pathway of Toxicity	Cognitive Outcome
Air pollution	Vehicle emissions Industrial factory sites Construction sites	White matter hyperintensities and demyelination Neuroinflammation	Learning disabilities Lowered IQ Memory and attention deficits
Lead	Lead-based paints Direct ingestion Maternal smoking Lead-based water pipes	Impeding synaptic transmission through mimicking of calcium Reduction of NMDA receptors Phosphorylation of PKC	Working memory deficits Lowered IQ ADHD
Chemical pesticides	Household Polluted neighborhoods	Reduction of cholinergic neurons	Impaired cognition Lowered IQ
Tobacco smoke	Maternal and household cigarette smoking	DNA methylation	Parent-reported learning disabilities
Polybrominated diphenyl ethers	Household products and appliances	Deficits and excesses in thyroid hormones Oxidative stress	Impaired memory Lowered IQ

## Data Availability

No new data were created or analyzed in this study. Data sharing is not applicable to this paper.
